# Potential role of herbal remedies in stem cell therapy: proliferation and differentiation of human mesenchymal stromal cells

**DOI:** 10.1186/s13287-016-0366-4

**Published:** 2016-08-11

**Authors:** Vindya Lankika Udalamaththa, Chanika Dilumi Jayasinghe, Preethi Vidya Udagama

**Affiliations:** Department of Zoology, Faculty of Science, University of Colombo, P.O. Box 1490, Colombo 03, Sri Lanka

**Keywords:** Stem cell therapy, Human mesenchymal stromal cells, Bone marrow derived, Proliferation and differentiation effectors, Synthetic stimuli, Herbal extracts, Standardization and quality control, Reparative and regenerative therapy

## Abstract

Stem cell therapy has revolutionized modern clinical therapy with the potential of stem cells to differentiate into many different cell types which may help to replace different cell lines of an organism. Innumerous trials are carried out to merge new scientific knowledge and techniques with traditional herbal extracts that may result in less toxic, affordable, and highly available natural alternative therapeutics. Currently, mesenchyamal stromal cell (MSC) lines are treated with individual and mixtures of crude herbal extracts, as well as with purified compounds from herbal extracts, to investigate the mechanisms and effects of these on stem cell growth and differentiation. Human MSCs (hMSCs) possess multilineage, i.e., osteogenic, neurogenic, adipogenic, chondrogenic, and myogenic, differentiation abilities. The proliferative and differentiation properties of hMSCs treated with herbal extracts have shown promise in diseases such as osteoporosis, neurodegenerative disorders, and other tissue degenerative disorders. Well characterized herbal extracts that result in increased rates of tissue regeneration may be used in both stem cell therapy and tissue engineering for replacement therapy, where the use of scaffolds and vesicles with enhanced attaching and proliferative properties could be highly advantageous in the latter. Although the clinical application of herbal extracts is still in progress due to the variability and complexity of bioactive constituents, standardized herbal preparations will strengthen their application in the clinical context. We have critically reviewed the proliferative and differentiation effects of individual herbal extracts on hMSCs mainly derived from bone marrow and elaborated on the plausible underlying mechanisms of action. To be fruitfully used in reparative and regenerative therapy, future directions in this area of study should (i) make use of hMSCs derived from different non-traditional sources, including medical waste material (umbilical cord, Wharton’s jelly, and placenta), (ii) take account of the vast numbers of herbal extracts used in traditional medicine globally, and (iii) investigate the mechanisms and pathways of their effects on hMSCs.

## Background

Stem cell therapy is at the forefront of regenerative medicine with the goal of regenerating and repairing injured tissues in the body. It is emerging as a promising therapeutic technique where tissues generated from stem cells are grafted on damaged tissue to repair and regenerate the original tissue [1]. In addition, stem cells are being investigated for their potential to produce whole organs for organ replacement. This rapidly evolving field of therapy has great promise in treating incurable diseases such as Parkinson’s disease [2], Alzheimer’s disease [3], and diabetes mellitus [4].

Stem cells possess the ability to self-renew and to differentiate into a plethora of different mature cell lineages under certain physiological or experimental conditions. Human mesenchymal stromal cells (hMSCs) are a multipotent group of cells that can be effortlessly isolated from a number of adult tissue types such as bone marrow, adipose tissue, and medical waste material such as umbilical cord and placenta [5]. These have the potential to differentiate into a wide variety of cell lineages (i.e., osteoblasts, adipocytes, chondrocytes, tenocytes, and myocytes [5]) with much promise for use in stem cell therapy. Due to these advantageous properties of MSCs, techniques for increasing MSC proliferation and differentiation are under much scrutiny. In existing stem cell therapy, extensive use of synthetic and semi-synthetic substances, i.e., recombinant cytokines and growth factors currently used as proliferative and differentiation factors in stem cell therapy, may lead to side effects and toxicity while being exorbitantly expensive. Due to these drawbacks, researchers are constantly searching for alternative natural products to be used as growth factors in stem cell therapy.

Herbal remedies have been used in traditional medicine practices for centuries for a wide range of diseases and are a promising alternative, offering substantial improvement of patient conditions and significantly decreased disease symptoms [6, 7], although the mechanisms of action of individual and mixtures of herbal plant extracts mostly remain undetermined. Investigating the proliferative, differentiation, and cytotoxic effects of different herbal extracts on stem cells may provide in-depth insights into their disease-curing mechanisms.

**Fig. 1:**
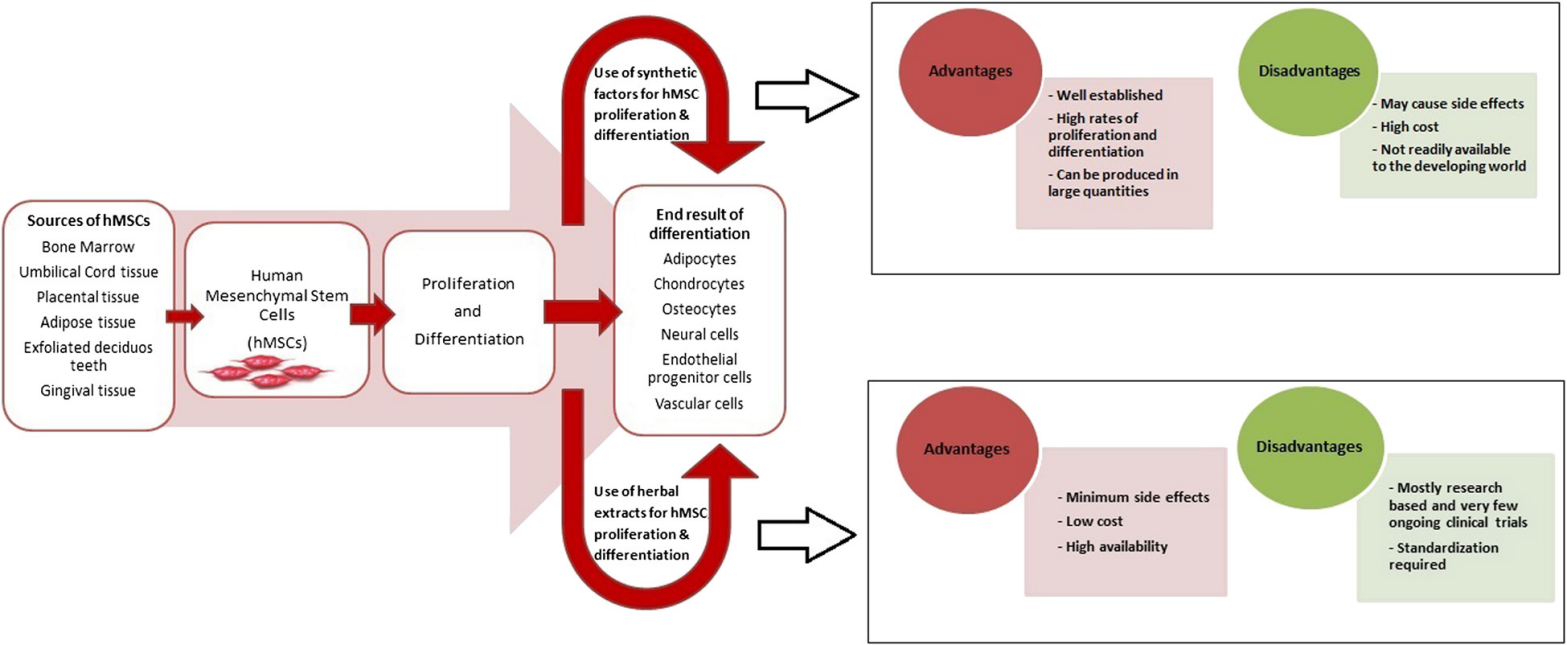
Human mesenchymal stromal cells (hMSCs) are isolated from different tissue types and can be proliferated and differentiated using both synthetic and herbal extracts into different cell types. Both synthetic and herbal effectors have their advantages and disadvantages in hMSC proliferation and differentiation

Low rejection rates, the availability and easy isolation of source cells, and in vitro culturing methods have made stem cell therapy advantageous over many other therapeutic methods. New technologies in the emerging field of regenerative medicine and stem cell therapy are currently under development. In the US, more than 100 companies invested nearly 850 million dollars in 2007 and over 55 products were in either clinical or preclinical trials [8]. The literature reviewed here is on recent investigations on the effects of different individual and mixtures of herbal extracts on the proliferation and differentiation of hMSCs, with their underlying mechanisms of action elucidated where possible (summarized in Fig. 1).

## Mesenchymal stromal cells

Because of their scientifically proven abilities and enormous clinical potential, MSCs are currently being researched extensively worldwide.

MSCs are a type of multipotent stem cell and are categorized as adult stem cells as they are isolated from adult tissues. They have the potential to differentiate naturally into adipocytes, osteocytes, and chondrocytes and can be induced into muscle, neurons, glia, and tendon-ligament [9]. MSCs reside in almost all postnatal human tissues and can be easily isolated from bone marrow (BM), umbilical cord (UC), umbilical cord blood (UCB), adipose tissue, dental pulp, cervical tissue, and muscle tissue [10]. MSCs have advantages over embryonic stem cells (ESCs), such as their easy isolation and in vitro culture and minimal ethical issues associated with their use, and, because of their HLA-DR (human leukocyte antigen-antigen D related)-negative characteristic, they are not recognized as foreign cells when used in therapy [9].

The in vitro culture and differentiation of hMSCs derived from BM was practiced in laboratories globally until recent years but sampling difficulties and age-dependent decreases in the number and differentiation potential of hMSCs led to the search for alternative sources [11]. UC and UCB are disposable biological material/medical waste at parturition and have been used in scientifically proven procedures to isolate hMSCs with the potential to differentiate into multipotent cell types [12, 13]. Due to the high availability of UC and UCB, hMSC requirements for transplantation could be fulfilled easily using them [14]. Extra-embryonic tissue-derived MSCs have demonstrated immune-privileged characteristics and broader multipotent plasticity [15]. UCB-hMSCs have demonstrated the highest rates of cell proliferation and clonality and significantly lower expression of p53, p21, and p16, well known markers of senescence, compared with BM-hMSCs and adipose tissue-derived (AD) hMSCs [16]. This has made UC- and UCB-derived hMSCs more popular than those derived from BM in recent years.

In 2015, 493 MSC-based clinical trials were recorded in the database of the US National Institutes of Health, those on bone and cartilage diseases being the highest proportion and cardiovascular diseases being the second highest [10]. hMSCs are currently used in clinical studies of tissue engineering [17], clinical engraftments to repair damaged tissues, and immune disorder therapy [18]. They also have potential applications in dentistry, metabolic disorders, disorders that result in tissue degeneration, and replacing damaged tissue due to accidents. Clinical trials for osteoporosis are being carried out using hMSCs derived from different sources such as BM, adipose tissue, and UC tissue by exogenous introduction of the stem cells into osteoporosis sites [19]. Research on diabetes have utilized MSCs to produce insulin-producing cells [20], treat diabetic ulcers and limb ischemia [21], and enhance islet engraftment and survival [22]. The potential of MSCs to regenerate heart and skeletal muscle tissue has been investigated in murine models, with results leading to preclinical trials on Duchenne muscular dystrophy to replace degenerating muscle tissue and also as carriers with a functioning dystrophin gene [23]. MSCs are candidates in cancer therapy as carriers for anticancer gene therapy [24] and have proven their ability as anti-tumor agents against Kaposi’s sarcoma [25], colon carcinoma [26], Lewis Lung carcinoma, and B16F10 melanoma [27].

## Current stimulants in stem cell therapy: cytokines and growth factors

Different semi-biological and synthetic substances are currently used for the proliferation and differentiation of MSCs. During the early scientific research, components isolated from human biological sources were used for this [28] but because of ethical controversies and the long and tedious downstream processing for the isolation and purification of such substances, recombinant and synthetic components have emerged as replacements. Currently, a cocktail of dexamethasone, ascorbic acid, and β-glycerophosphate is used for in vitro osteogenic differentiation of hMSCs, while 3-isobutyl-1-methylxanthine, hydrocortisone, and indomethacin are used for adipogenic differentiation, and fibroblast growth factor and bone morphogenetic protein (BMP)-2 are used for higher rates of proliferation [12, 29]. Several extracellular matrix proteins have demonstrated proliferation and differentiation potential-preserving properties [29]. A cytokine, granulocyte colony stimulating factor (G-CSF), has been used to mobilize and differentiate murine MSCs into cardiomyocytes [30].

### Drawbacks of current stimulants

Many companies produce recombinant cytokines using *Escherichia coli* cells, plant cells, and other mammalian cells. Yet, the recombinant and synthetic cytokines, growth factors, and other proteins involved in the proliferation and differentiation of stem cells used in cell culture and in clinical treatment may show side effects and toxic effects [31] when used continuously and the stem cells may have the potential for rejection due to the different origins of the stimulants. Ascorbic acid used for osteogenic differentiation is unstable at 37 °C and toxic at high doses [32]. Dexamethasone shows immunosuppressive properties, which limit its application in osteoblast differentiation [33], and some growth factors can cause malignant formation in cells [34]. Most importantly, these reagents rapidly degrade and thus need to be continuously replaced and are highly expensive, making them unaffordable in developing countries. Due to these restrictions, a new research stream has evolved to investigate the use of natural products as effective stem cell proliferation and differentiation stimulants with minimum side effects, low toxicity, and high availability and affordability.

## Herbal remedies

Herbal extracts have shown much promise in the proliferation and differentiation of hMSCs in many different studies. The origin of these herbal extracts is mainly from Chinese traditional medicine, Indian Ayurveda medicine, and other South East Asian and Middle Eastern traditional medicine practices. Herbal extracts contain a plethora of phytochemicals such as polyphenols, flavonoids, and other plant-derived chemicals which synergistically aid in treating diseases in traditional medicine methods. Not only individual herbal extracts but also mixtures of different herbal extracts have shown promising results in traditional medicine. Also, the different parts of medicinal herbs, such as roots, leaves, stem, and fruits, are used in preparations for the treatment of different diseases. The herb *Tithonia diversifolia* is a worthy example, an extract of it being used for the treatment of diabetes, diarrhea, menstrual pain, malaria, hematomas, hepatitis, hepatomas, and wound healing [35]. The proven ability of many herbal extracts to treat a range of diseases has captured the attention of modern scientists and preliminary research is being carried out using stem cells and other cell types to find herbal extracts that are suitable stimulants based on their promising results in traditional medicine. Since herbs grow naturally, their local availability is high and the preliminary production costs will presumably be lower than for recombinant growth factors. As these extracts are composed of naturally occurring medicinal herbs, which may be regularly consumed by local communities, these may cause minimum side effects and have lower toxicity than the current stimulants. Therefore, herbal remedies may be safe and affordable alternatives to highly expensive recombinant and synthetic stimulants.

### Effects of herbal extracts on the differentiation and proliferation of hMSCs

The studies described in the following sections elaborate on such herbal remedies and their possible mechanisms of action on hMSCs.

#### Osteogenic effects of herbal extracts

A traditional Chinese herbal formula (ZD-1) was found to have stimulatory effects on the proliferation and inhibitory effects on mineralization of hMSCs through down-regulation of several osteogenic markers such as osteocalcin, BMP-2, and osteopontin in the late stages [36].

The dried root of *Drynariae fortune*, Rhizoma Drynariae, contains flavonoid and triterpenoid compounds and its use results in increased bone cell viability, intracellular total proteins, alkaline phosphatase, and acid phosphatase [37]. Naringin, which is the main component of the Rhizoma Drynariae extract, enhanced the proliferation and osteogenic differentiation of BM-derived hMSCs [38]. The regulation of β-catenin, which is involved in osteoblastogenesis via Akt (protein kinase B) and AMPK (AMP activated protein kinase) signaling, has been demonstrated as a possible mechanism for the osteogenic properties of naringin [39].

An ethanol extract of the fruit of *Ligustrum lucidum* demonstrated an inhibitory activity on the proliferation of BM-derived hMSCs in a dose-dependent manner and a cytotoxic effect at a concentration of ≥200 μg/mL [40]. Conversely, the same study illustrated accelerated osteogenic activity at two specific concentrations, 50 μg/mL and 75 μg/ml. A suggested mechanism of action is based on a significant increase in expression of osteogenesis stimulating genes, β-catenin, BMP-2, cyclin D1, MT1-MMP (membrane type-1 matrix metalloproteinase), osteoprotegerin, and TBX3(T-Box 3) [40].

Dried root extract of the Korean herb *Dipsacus asper*, Dipsaci Radix, is used in the treatment of bone fracture. A dichloromethane fraction of Dipsaci Radix demonstrated not only that the whole extract possessed the ability to enhance osteoblastic differentiation from BM-derived hMSCs, but that the isolated single compound (hedraganin-3-O-(2-O-acetyl)-α-L-arabinopyranoside) worked similarly. Researchers suggested that the enhanced osteoblast differentiation was due to induced alkaline phophatase activity and expression of bone-specific proteins (bone sialoprotein and osteocalcin) by both the crude Dipsaci Radix extract and the isolated compound [33].

*Foeniculum vulgare* has been used in traditional medicine to increase milk secretion, promote menstruation, facilitate birth, and alleviate the symptoms of dysmenorrhea, which are conditions involving estrogenic activity. Based on estrogenic activity, a study on an ethanol extract of *F. vulgare* dried root showed a significant increase of BM-derived hMSC proliferation and differentiation into osteoblasts. The antiosteoporotic activity was thought to be stimulated through estrogenic activity of trans-anatole, an abundant component of the *F. vulgare* extract [41].

*Herba epimedii* extract showed increased rates of osteogenic activity on human BM-derived hMSCs via enhanced mRNA expression of BMP-2, BMP-4, Runx2 (Runt-related transcription factor 2), beta-catenin, and cyclinD1, all of which are BMP or Wnt signaling pathway-related regulators. This elucidated that the flavonoids of this extract promote osteogenesis through the BMP or Wnt-signaling pathway [42].

*Ferula gummosa* is used in Iranian traditional medicine and has applications in treating different types of diseases. The ethanol root extract of *F. gummosa* was proven to proliferate and differentiate human BM-derived hMSCs into osteocytes, demonstrating increased alkaline phosphatase activity [43].

#### Anti-adipogenic effects of herbal extracts

A Chinese herbal remedy, Quzhisu, had inhibitory effects on the adipogenic differentiation of BM-derived hMSCs by reducing the expression of PPARγ (peroxisome proliferator activated receptor γ) mRNA [44].

*Tithonia diversifolia* demonstrated antioxidant properties, which may help in treating obesity, a condition associated with decreased antioxidant levels resulting in high levels of adipogenesis [35]. This study recorded significant antiadipogenic activity due to reduced levels of reactive oxygen species by treating adipose derived hMSCs with a *T. diversifolia* aqueous extract [35]. Increased levels of pAMPK (phosporylated 5′-adenosine monophoshate-activated protein kinase), a key regulating enzyme involved in adipocyte differentiation and maturation [45], were detected [35].

Aloe-emodin is an anthraquinone present in aloe latex and has proven inhibitory effects on adipocyte differentiation when hMSCs were induced with 3-isobutyl-1-methylxanthine (IBMX) for adipogenesis. In comparison with control cultures, hMSCs treated with aloe-emodin had reduced expression levels of mRNA for resistin, adiponectin, aP(2), lipoprotein lipase, PPARγ, and tumor necrosis factor-α, which influence adipogenic pathways [46].

#### Neurogenic effects of herbal extracts

BM-derived hMSCs treated with a 1 % acetic acid extract of *Mucuna gigantea* had high proliferative characteristics and exhibited higher expression of mRNA for nestin (a neural precursor marker) and β-III tubulin (an immature neuron marker), suggesting the importance of *M. gigantea* in neural differentiation. As the *M. gigantea* extract contains L-DOPA, which is a precursor for neurotransmitter dopamine, using it in nerve-related stem cell therapy would enhance treatment capacity [47].

The extract of *Angelica sinensis* (Danggui) dried root, known as Radix Angelica sinesis (RAS), decreased β-amyloid peptide-induced neurotoxicity and tau phosphorylation in cultured cortical neurons [48], suggesting it has applications in neurodegenerative disorders. Treatment with RAS extract showed significantly higher percentages of neural like cell differentiation from AD-hMSCs compared with hMSCs treated with butylated hydroxyanisole; a commonly used neuronal inducer [49]. Ferulic acid, a main component of the RAS extract, also has proven ability to inhibit neurotoxic β-amyloid peptide aggregation in animal models [50], but the effects on patients with neurodegenerative disorders are yet to be determined.

*Salvia miltiorrhiza* extract demonstrated positive effects on differentiating Wharton jelly-derived hMSCs into neural like cells with significant morphological changes. Strongly positive markers were observed for nestin, β-tubulin, neurofilament, and glial fibrillary acidic protein. Neurite outgrowth-promoting protein expression (a neural cell marker) was markedly increased in hMScs after induction with the *S. miltiorrhiza* extract [51].

#### Endothelial/vascular genesis and angiogenesis effects of herbal extracts

Olive leaf extract resulted in hMSCs differentiating into endothelial cells and further into tubular structures, which are required in angiogenesis and vasculogenesis processes, while the genes for vascular endothelial growth factor, PCAM, platelet derived growth factor receptor, and vascular endothelial growth factor receptor (VEGFR)-1, involved in endothelial differentiation, are increasingly expressed in olive leaf extract-treated cells [52].

The rhizome extract of *Curcumin longa L* contains the phytochemical curcumin as the main component, which has antioxidant and anti-inflammatory properties. As stem cells require an antioxidant mechanism to survive and repair cells, curcumin with its antioxidant properties becomes a potential candidate to be applied in stem cell therapy research. An ethanol extraction of curcumin dried rhizome resulted in enhanced proliferation and differentiation of AD-hMSCs into endothelial progenitor cells through increased expression of the cell surface markers CD34, CD133, and VEGFR2 [53].

#### Proliferative and other effects of herbal extracts

*Dhanwantram kashaya*, used in Ayurvedic medicine to stimulate growth and nerve regeneration, resulted in increased proliferation and delayed senescence of Wharton jelly-derived hMSCs [54]. Yet another Chinese herbal remedy, Dan-Qi-TongMai-Pian, prepared mainly using *Astragalus* and *Salvia*, resulted in negative apoptosis of BM-derived hMSCs via stimulating c-IAP-1/2 expression and restricting caspase-3 activation [55].

The leaf extract of *Carica papaya* was found to have an effect on improving thrombocyte counts in both human and murine models, which suggest *C. papaya* leaf extract as an invaluable potential therapeutic agent for diseases such as dengue. The *C. papaya* extract up-regulated the in vitro synthesis of thrombopoiesis-related cytokines interleukin-6 and stem cell factor by hMSCs isolated from exfoliated deciduous teeth [56].

*Viscum album*, also known as Korean mistletoe lectin, was investigated for cytotoxic and proliferative activities on placenta-derived hMSCs. *V. album* extract showed cytotoxicity on HepG2 cancer cells at low concentrations of 1–5 pg/ml but induced significant proliferative properties in naive placenta-derived hMSCs via autophagy mechanisms [57].

#### Beneficial effects of herbal extracts in scaffolds

In vivo studies have shown an extract of *Cissus quadrangularis*, known as the *Asthisandhani* (bone setter) in Indian traditional medicine, to have bone fracture-healing properties [58]. Scaffolds treated with a *C. quadrangularis* extract showed significant differences with regard to hMSC proliferation, attachment, and enhanced osteoblast differentiation properties compared with scaffolds not treated with the extract [59].

The extract of *Terminalia bellirica* contains gallic acid, with anti-oxidant and cytoprotective properties, as a major component. In a study on hydrogel composition for use in stem cell therapy, a *T. bellirica* extract was found to result in significantly higher rates of hMSC proliferation and cell attachment [60].

#### Adverse effects of herbal extracts

The root extract of *Angelicae dahuricae* has been used as an anti-inflammatory, analgesic, antipyretic, and antioxidant remedy in Chinese and Korean herbal medicine. Nevertheless, this did not produce any significant rates of proliferation in hMSCs derived from gingiva [61].

The dried root of *Cimicifuga heracleifolia* or *Cimicifuga foetida* is known as Cimicifugae Rhizoma. An extract of Cimicifugae Rhizoma possesses anti-inflammatory, analgesic, and antipyretic properties and also been suggested as a potential treatment for dental diseases. hMSCs derived from healthy gingival tissue were treated with an aqueous Cimicifugae Rhizoma extract which, at high concentrations (such as 100 and 1000 μg/ml) affected stem cell morphology and reduced cell viability, suggesting potential adverse effects if administered orally [62].

A study on a root extract of *Asiasarum*, suggested for treating oral diseases, showed reduced cell viability of hMSCs derived from gingiva at higher concentrations (such as 100 and 1000 μg/ml), suggesting adverse effects on oral tissues at high doses of the extract [63].

### Current drawbacks of use of herbal extracts

Herbal extracts may show promising results in in vitro studies but to apply these natural products in stem cell therapy will require more quality research to provide a better understanding into their mechanisms of action and effective pathways. As a crude extract a herbal could display beneficial properties, but its efficacy may be reduced during the manufacturing processes when produced at a commercial scale. Also, different solvents used to prepare crude extracts may cause adverse effects when used in therapy. Decreased absorption problems may occur when administered orally or intravenously; hence, local administration would be required when transplantation is performed, which can result in an invasive, painful procedure for the patient.

Although herbal products are endorsed as an excellent alternative to synthetic interventions, clinical applications are challenging due to the variability and complexity of bioactive constituents present in herbal formulations. Genetic, environmental, and cultural factors as well as the process of preparation affect the quality and the quantity of bioactive constituents, which may lead to undesirable side effects and low bioactivities. A well-defined and constant composition of the active constituents is a prerequisite for clinical application. Hence, “standardization”, which entails a set of scientific evaluations that assure quality, efficacy, safety, and reproducibility, was introduced to ensure the safety of herbal formulations in the global market [64]. Proper standardization will minimize quality issues with herbal preparations and ensure safe application of these in stem cell therapy.

## Conclusion

Stem cell therapy has immense potential in clinical use when linked with the differentiation and proliferative properties of herbal extracts. Cutting edge technology and widening knowledge in medicine have led researchers to investigate the pathways and regulatory effects of herbal extracts on stem cells, which have shown positive effects in a wide range of diseases.

According to the literature reviewed, herbal extracts as stimulants of hMSC proliferation and differentiation show outstanding results and are suitable for further investigations on their chondrogenic, osteogenic, vasculogenic, and neurogenic potential. The proliferative and differentiation activities of hMSCs treated with herbal extracts may provide for future therapeutics and tissue replacements for diseases such as osteoporosis, heart disease, Parkinson’s disease, and cartilage replacement. Herbal extracts may also be used to treat attaching gels and scaffolds in regenerative therapy. Nevertheless better insights into their mechanisms and modes of action should be studied using hMSCs as most studies have so far been restricted to in vitro and in vivo studies of murine models.

Most of the studies reviewed here were aimed at osteogenic differentiation and proliferation of BM-derived hMSCs; the reason for the extensive use of BM-derived hMSCs in research is their well characterized nature and the wide experience with these cell lines. Nonetheless, studies on hMScs derived from non-traditional sources, such as AD-hMSCs and those from medical waste at parturition, such as UC-, UCB-, and Wharton jelly-derived hMSCs, and placental hMSCs, are being explored since these are associated with fewer ethical considerations than other stem cells and they can be obtained using easy sampling methods. Use of embryos for ESC culture is bound by heavy ethical regulations and religious issues globally. hMSCs are mainly isolated from surgical and parturition waste material, so use of these biological sources does not concern donors to a greater extent, whereas donors are highly concerned about the use of embryos in ESC procedures.

As most of the discussed stimulants are crude extracts of medicinal herbs, once standardized, producing these at a commercial scale will be highly cost effective compared with a recombinant, synthetic line of stimulants. Also, production lines could be established globally, with medicinal herbs grown locally in different regions. Once their availability becomes high and costs decrease, stem cell therapy research and clinical applications will be affordable worldwide, which would help to increase disease-treating capabilities using stem cell therapy. Legal complications may arise due to the use of indigenous herbal varieties in foreign production lines, but a globally accepted agreement may successfully address this issue. Several herbal medicines, including *Curcuma longa* and *Salvia*, discussed in this review have beneficial immunomodulatory properties which would boost the use of certain herbals in clinical applications [65]. This review aims to direct stem cell researchers to a potentially effective novel world of stimulants which is currently in its infancy but may pave the way as invaluable candidates in future regenerative and reparative clinical applications with further research.

Strategic use of herbal remedies as stimulants of proliferation and differentiation in stem cell therapy may result in cost-effective, highly available, non-toxic alternatives for therapeutic application as there are vast number of herbal extracts used in traditional medicine globally. Well standardized herbal preparations with consistent quality and effects will be a potential breakthrough in stem cell therapy.

## Abbreviations

AD, adipose derived; AMPK, AMP activated protein kinase; BM, bone marrow; BMP, bone morphogenetic protein; ESC, embryonic stem cell; hMSC, human mesenchymal stromal cell; MSC, mesenchymal stromal cell; PPARγ, peroxisome proliferator activated receptor γ; RAS, Radix Angelica sinesis; UC, umbilical cord; UCB, umbilical cord blood; VEGFR, vascular endothelial growth factor receptor
